# Subendocardial stress in pre‐eclampsia

**DOI:** 10.1111/anec.12769

**Published:** 2020-06-05

**Authors:** Tomio Tran, Morteza Farasat, Mori J. Krantz

**Affiliations:** ^1^ Department of Medicine University of Colorado School of Medicine Aurora CO USA; ^2^ Department of Medicine Division of Cardiology Denver Health and Hospital Authority Denver CO USA

**Keywords:** basal septal bulge, left ventricular outflow obstruction, myocardial infarction, pre‐eclampsia, pregnancy, subendocardial ischemia

## Abstract

A primigravida 26‐year‐old woman who had developed pre‐eclampsia with malignant hypertension at 30 weeks of gestation suffered acute myocardial infarction two days postpartum. Electrocardiogram demonstrated diffuse ST‐segment depression suggestive of subendocardial ischemia. Echocardiography demonstrated focal asymmetric left ventricular hypertrophy, with a characteristic “basal septal bulge”, and a left ventricular mid‐cavitary gradient of 51 mmHg. Coronary angiography revealed normal coronary arteries and vascular flow. Peripartum acute myocardial infarction is rare and portends a high mortality. However, to date, only one case of acute myocardial infarction associated with asymmetric left ventricular hypertrophy and pre‐eclampsia has been described in the literature.

## 1 CASE DESCRIPTION

A 26‐year‐old primigravida woman presented to obstetrics clinic at 30 weeks of gestation to establish prenatal care after moving to the United States from Ghana one week earlier. At that time, she complained of severe headaches and bilateral lower extremity edema. She had been prescribed methyldopa and nifedipine at the beginning of her pregnancy for hypertension, but denied a past personal or family history of cardiovascular disease. Physical examination was remarkable for a blood pressure of 216/133 mmHg and bilateral lower extremity pitting edema, and laboratory findings included an elevated serum creatinine level of 2.1 mg/dl (normal 0.5–1.39 mg/dl) with nephrotic‐range urinary protein/creatinine ratio at 11.4 (normal < 0.3), leading to a diagnosis of pre‐eclampsia with severe features. Given the presence of malignant hypertension, she was admitted to the intensive care unit. However, despite therapy with maximal doses of intravenous labetalol and hydralazine, and oral nifedipine, her blood pressure remained uncontrolled. Fetal ultrasound showed severe intrauterine growth restriction and fetal monitoring demonstrated fetal distress, manifest by a category II tracing with deep variable decelerations and periods of minimal variability. Given the high‐risk fetal tracing and maternal refractory malignant hypertension, delivery by Cesarean section was recommended, which was carried out without complications.

**Figure 1:**
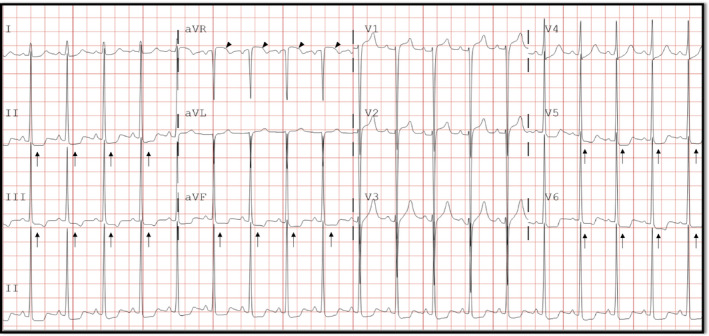
12‐lead ECG demonstrates diffuse ST‐segment depression (arrows) with ST‐segment elevation in lead aVR (arrowheads)

**Figure 2:**
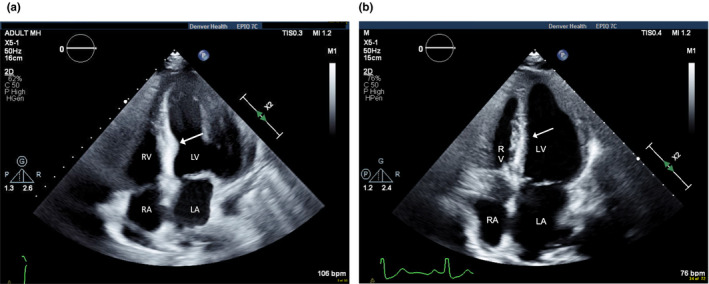
Initial Transthoracic echocardiotraphy (a) demonstrates basal septal bulge (arrow), which resolved (b) after treatment of hypertension. RV = right ventricle, RA = right atrium. LA = left atrium. LV = left ventricle

Two days postpartum, while blood pressure remained elevated at 191/105 mmHg, the patient became acutely diaphoretic and mildly dyspneic, but denied chest pain. Electrocardiogram (ECG) showed left ventricular hypertrophy (LVH) by Sokolow‐Lyon criteria with ST‐segment depressions in leads II, III, aVF, V5, and V6 and ST‐segment elevation in lead aVR (Figure 1). Blood work was remarkable for an elevated troponin‐I level of 4.63 ng/ml (normal < 0.03 ng/ml), consistent with acute myocardial infarction (AMI). Physical examination revealed tachycardia with a regular rhythm and a III/VI holosystolic murmur heard best at the apex, without jugular venous distention or lower extremity edema. Computed tomography did not reveal any evidence of pulmonary embolism. Transthoracic echocardiography demonstrated severe LVH with a prominent bulge at the basal septum (Figure 2a), a mid‐cavitary gradient of 51 mmHg, but no segmental wall motion abnormalities. Cardiac catheterization revealed an elevated left ventricular end‐diastolic pressure (LVEDP) of 30 mmHg, an intracavitary gradient of 15 mmHg, but no coronary artery disease. Labetalol was discontinued in favor of carvedilol, which is indicated in AMI, and antihypertensive medications were increased with improvement of her blood pressure and troponin‐I levels.

Two months postdischarge, the patient had a repeat echocardiogram. Her blood pressure at this time was 139/105 mmHg. Moderate LVH was present; however, the intracavitary gradient and characteristic “basal septal bulge” seen on the previous echocardiogram were no longer present (Figure 2b).

## 2 DISCUSSION

This patient demonstrated the characteristic findings of pre‐eclampsia with severe features (Magee et al., 2014) and hypertensive emergency, both of which share the diagnostic criteria of elevated blood pressure associated with end‐organ dysfunction (Whelton et al., 2018). The patient also met the criteria for AMI, given clinical, ECG and cardiac biomarker profiles (Thygesen et al., 2018). Although AMI during pregnancy is rare, occurring at a rate of approximately 8.1 per 100,000 hospitalizations according to an analysis of a US national sample, it carries an approximately 40‐fold increase in mortality risk (Smilowitz et al., 2018).

The differential diagnosis for AMI during pregnancy includes acute atherothrombotic coronary artery disease (type 1 AMI), coronary vasospasm, spontaneous coronary artery dissection (SCAD), and type 2 AMI secondary to a mismatch in oxygen supply and demand. The presence of ST‐segment elevation in lead aVR with diffuse ST‐segment depressions has been shown to be a strong predictor of both left main and three‐vessel coronary artery disease (D'Ascenzo et al., 2012). Therefore, type 1 AMI and SCAD were prioritized in this case. The true incidence of type 1 AMI during pregnancy is unknown; however, 25% of AMI patients underwent coronary revascularization in an analysis of a US national sample (Smilowitz et al., 2018). In the same analysis, SCAD was identified in 15% of patients (Smilowitz et al., 2018), though in a previous case‐series, SCAD was found to be the leading etiology of AMI in pregnancy, comprising of 43% of patients (Elkayam et al., 2014). Clinching a diagnosis of SCAD requires a high index of suspicion and coronary angiography. It should be noted, however, that invasive coronary angiography may result in further extension of the coronary artery dissection (Hayes et al., 2018), and intervention should generally be deferred if possible. As such, given the absence of acute atherothrombotic disease and SCAD, type 2 AMI secondary to very high wall stress, augmented by the intracavitary gradient, was considered the most likely diagnosis in this patient, although coronary vasospasm cannot be definitively excluded, especially considering that endothelial dysfunction is a hallmark of pre‐eclampsia (Melchiorre, Sharma, & Thilaganathan, 2014).

In a case‐control study, women with pre‐eclampsia who had echocardiography performed showed cardiac remodeling, which included concentric LVH, diastolic dysfunction, and increased cardiac work indices (Melchiorre, Sutherland, Baltabaeva, Liberati, & Thilaganathan, 2011). Mid‐cavitary obstruction can be seen in a subgroup of patients with hypertrophic cardiomyopathy and may be observed with concomitant apical aneurysm, which was absent in our patient (Efthimiadis et al., 2013). Our patient's echocardiogram also showed focal, basal septal hypertrophy, described as a characteristic “basal septal bulge,” similar in appearance to the “sigmoid septum” that is often seen in the elderly, and can confound the diagnosis of hypertrophic cardiomyopathy (Canepa et al., 2016). However, this finding has also been described in patients with pre‐eclampsia (Melchiorre et al., 2011). Increased left ventricular wall thickness is an adaptive response, aiming to reduce wall stress, which can result in increased LVEDP, which in turn can reduce coronary perfusion pressure.

Coronary artery perfusion pressure (CPP) is the difference between aortic diastolic blood pressure and LVEDP. Normally, CPP is auto‐regulated between approximately 60 and 180 mmHg. However, patients with LVH have been shown to require higher blood pressures to maintain adequate CPP (Cruickshank, 1992). Left heart catheterization in our patient revealed a CPP of 52 mmHg, which may have been inadequate to perfuse the subendocardial tissue, thus resulting in subendocardial ischemia, which would not be expected to result in segmental wall motion abnormalities. Therefore, we contend that the etiology of AMI in our patient was subendocardial ischemia due to increased oxygen demand in the context of severe LVH and very high afterload from malignant hypertension, coupled with reduced coronary artery perfusion pressure secondary to left ventricular outflow tract (LVOT) obstruction and malignant hypertension, both of which increase LVEDP.

Aggressive blood pressure control is the mainstay of treatment in hypertensive crises and pre‐eclampsia. However, in rare cases of LVOT obstruction, CPP may be further diminished. Reducing chronotropy is critical in such cases as increasing the duration of diastole and reducing LVEDP can lead to improved CPP. First‐line treatment in hypertensive crises of pregnancy include labetalol, methyldopa, and nifedipine, with hydralazine used as a second‐line agent (Regitz‐Zagrosek et al., 2018). While labetalol is the preferred beta‐antagonist in pregnancy, its beta‐antagonistic activity on reducing chronotropy is minimal (MacCarthy & Bloomfield, 1983) and it has not been utilized in the setting of AMI. Beta‐antagonists carry the risk of causing intrauterine growth restriction (IUGR); however, its use in pregnancy is warranted if the benefits outweigh the risks. In such cases, beta‐1‐selective antagonists such as metoprolol and bisoprolol are preferred, while atenolol should be avoided (Regitz‐Zagrosek et al., 2018). Nonselective beta‐antagonists should generally be avoided, however, carvedilol may be an acceptable agent since a small retrospective study showed that pregnant women receiving carvedilol displayed no IUGR (Tanaka et al., 2016).

To our knowledge, this is only the second report of peripartum AMI associated with LVH and the previously reported, “basal septal bulge.” In the first published such case report, the patient was found to have a gene mutation associate with hypertrophic cardiomyopathy (Singla, Lipshultz, & Fisher, 2011). Our case highlights the maladaptive cardiac remodeling that occurs with pre‐eclampsia, which may predispose such patient to AMI via a unique, nonthrombotic mechanism. Specifically, this highlights the potential synergistic impact of LVH and LVOT obstruction, which in extreme cases, can result in subendocardial injury. In addition, presence of the characteristic “basal septal bulge” on echocardiography during the peripartum period, and its absence on follow‐up echocardiography, suggests that this may be a reversible finding in pre‐eclampsia.

## CONFLICT OF INTEREST

The authors declare that they have no conflicts of interest.

## AUTHOR CONTRIBUTION

Reviewed and approved the report: all authors; Wrote the main manuscript: Tran, Krantz, Farasat; Directed the report: Krantz; Collected data: Tran; Gave suggestions on the report: Farasat; Statistical analysis: NA.

## ETHICS

The report was conducted in accordance with the declaration of Helsinki and in accordance with the Colorado Multiple Institutional Review Board policies.
